# Atg9 is required for intraluminal vesicles in amphisomes and autolysosomes

**DOI:** 10.1242/bio.013979

**Published:** 2015-09-09

**Authors:** C. A. Bader, T. Shandala, Y. S. Ng, I. R. D. Johnson, D. A. Brooks

**Affiliations:** Mechanisms in Cell Biology and Diseases Research Group, School of Pharmacy and Medical Science, University of South Australia, Adelaide, South Australia 5001, Australia

**Keywords:** Atg9, Autophagy, Autophagosome, Amphisome, Autolysosome, Multivesicular endosome, Lysosome, Intraluminal vesicles

## Abstract

Autophagy is an intracellular recycling and degradation process, which is important for energy metabolism, lipid metabolism, physiological stress response and organism development. During *Drosophila* development, autophagy is up-regulated in fat body and midgut cells, to control metabolic function and to enable tissue remodelling. Atg9 is the only transmembrane protein involved in the core autophagy machinery and is thought to have a role in autophagosome formation. During *Drosophila* development, Atg9 co-located with Atg8 autophagosomes, Rab11 endosomes and Lamp1 endosomes-lysosomes. RNAi silencing of *Atg9* reduced both the number and the size of autophagosomes during development and caused morphological changes to amphisomes/autolysosomes. In control cells there was compartmentalised acidification corresponding to intraluminal Rab11/Lamp-1 vesicles, but in Atg9 depleted cells there were no intraluminal vesicles and the acidification was not compartmentalised. We concluded that Atg9 is required to form intraluminal vesicles and for localised acidification within amphisomes/autolysosomes, and consequently when depleted, reduced the capacity to degrade and remodel gut tissue during development.

## Abstract (precis)

**Summary:** The disappearance of intraluminal vesicles in amphisomes/autolysosomes upon Atg9 depletion suggests that Atg9 has a specific role in intraluminal vesicle formation in autophagic compartments.

## INTRODUCTION

Macroautophagy or autophagy is an intracellular process that is highly conserved from yeast through to mammals, and involves the encapsulation of cytoplasmic components into a double membrane structure called an autophagosome (Ohsumi, 2014). Autophagosomes can then undergo a sequential maturation process by interacting with endosomes to form amphisomes and then lysosomes to generate autolysosomes; which eventually results in the degradation of engulfed cytoplasmic material (Gordon and Seglen, 1988; Berg et al., 1998; Lamb et al., 2013a; Shen and Mizushima, 2014). This catabolic process is an important mechanism for the bulk degradation of cytoplasmic constituents, the clearance of protein aggregates, the recycling of aged or defective organelles and for combatting intracellular pathogens (Mizushima et al., 2008). Autophagosomes also have critical functional roles in cellular homeostasis, being specifically involved in energy/nutrient sensing, glucose/glycogen metabolism, and lipid transport, storage and metabolism (Singh et al., 2009; Singh and Cuervo, 2011). Autophagy operates at a basal level in most cell types, but can be specifically induced in response to hormonal and developmental signalling, nutrient restriction, aberrant protein folding, altered homeostasis/physiological stress, and various pathological conditions including neurodegenerative disease, infection, aging and cancer (Kundu and Thompson, 2008; Deretic, 2009; Kroemer et al., 2010; Liu and Ryan, 2012). While the core molecular machinery involved in autophagy has been identified (Tsukada and Ohsumi, 1993; Thumm et al., 1994; Harding et al., 1995), the mechanisms orchestrating this critical intracellular process and the precise role for each component of the molecular machinery are yet to be fully defined.

Over 30 autophagy related genes (Atg) have been discovered, mainly from studies in yeast (Tsukada and Ohsumi, 1993; Nakatogawa et al., 2009). Upon the induction of autophagy, by for example starvation, most of these Atg proteins are localised to a perivacuolar structure called the pre-autophagosome structure (PAS; Suzuki et al., 2001, 2007). The PAS is a precursor for autophagosome formation and involves the recruitment of the kinase complexes Atg1/ULK1 and Atg6/Beclin1 to a structure that generates an autophagosome-specific pool of phosphatidylinositiol-3-phosphate (Rubinsztein et al., 2012). This lipid pool drives the nucleation of the phagophore and recruitment of other autophagy-related proteins to the isolation membrane (Lamb et al., 2013b; Wirth et al., 2013). Membrane expansion and closure of the phagophore involves the Atg5-Atg12 complex, which acts as an E-3 ligase to mediate the lipidation of Atg8/LC3, and the latter remains associated with the outer autophagosome membrane during maturation (Klionsky and Schulman, 2014). The multiple-spanning integral membrane protein Atg9 is an early target of the Atg1/ULK1 kinase complex and Atg9 phosphorylation is required for the efficient recruitment of Atg8 and Atg18 to the site of autophagosome formation (Papinski et al., 2014); making it an essential component of the autophagic molecular machinery.

The integral membrane properties of Atg9 and its detection in different membrane compartments, including small vesicles that reside in close proximity to the Golgi, mitochondria and the PAS, have led to the suggestion that Atg9 is involved in membrane delivery to the expanding phagophore (Mari et al., 2010; Lamb et al., 2013b). In yeast, for example, Atg9 can be detected on small 30–60 nm vesicles at the PAS (Yamamoto et al., 2012) and may interact with the Atg17 scaffolding complex assembled by Atg1/ULK1 to facilitate Atg9 vesicle fusion (Sekito et al., 2009; Ragusa et al., 2012). This vesicular fusion may provide a membrane platform on which the isolation membrane can then be formed (Yamamoto et al., 2012; Shibutani and Yoshimori, 2014). In mammalian cells, Atg9 also localises with Atg1/ULK1 and Atg16L at recycling endosomes (Longatti et al., 2012; Puri et al., 2013) and is trafficked to the recycling endosome from the plasma membrane, possibly through the early endosome (Ravikumar et al., 2010; Longatti et al., 2012; Puri et al., 2013). While Atg9 might have a role in the formation of membrane platforms, it is sequestered to but not integrated into autophagosomes (Orsi et al., 2012), raising concerns over the ideas on membrane recruitment and suggesting that it has an alternate functional role.

Atg9 co-locates with endosomes following the induction of autophagy (Young et al., 2006; Tang et al., 2011), adding confusion about the precise role of Atg9 and leading to speculation about its involvement in autophagosome maturation. Amphisome formation, involving the heterotypic fusion of autophagosomes and endosomes, is known to utilise a range of vesicular machinery including, Rab11 (Savina et al., 2005; Fader et al., 2008), the SNARE protein VAMP3 (Fader et al., 2009) and the ESCRT proteins Vps 28, Vps25, Vps32 and Deep Orange (Lindmo et al., 2006; Rusten et al., 2007; Vaccari et al., 2009; Spowart and Lum, 2010). However, the exact involvement of this vesicular machinery and the regulatory mechanism is not clear. For example, while Rab11 is a marker for recycling endosomes it is also detected on multivesicular endosomes, and is involved in amphisome formation (Fader et al., 2008; Richards et al., 2011). In addition, Rab11 has a role in Atg9 trafficking from the plasma membrane to autophagic compartments (Ravikumar et al., 2010; Longatti et al., 2012; Puri et al., 2013). This trafficking of Atg9 by Rab11 compartments appears to be vital for autophagosome initiation, but has not been fully investigated in relation to amphisome formation (Ravikumar et al., 2010; Longatti et al., 2012; Puri et al., 2013). Other autophagy initiators also have functions in autophagosome maturation; for example, the Atg12-Atg5 initiation complex (part of the Atg12-Atg5-Atg16 complex), which is involved in autophagosome maturation via an interaction with the tethering protein TECRP1 during autolysosome formation (Chen et al., 2012). Lysosomal fusion with the maturing autophagosome to form autolysosomes, also involves specific vesicular machinery, including Rab7 and the SNARE protein Syntaxin 17, which binds to SNAP-29 and VAMP7 to form a ternary complex (Fader et al., 2009; Itakura et al., 2012; Takats et al., 2013). As the only integral membrane protein in the core autophagy machinery, Atg9 may play a role in endosome and lysosome recruitment, acting to facilitate vesicular fusion in manner similar to that proposed for its role in membrane recruitment to the phagophore (Takahashi et al., 2011).

*Drosophila* provides an ideal model system to investigate the role of Atg9 in autophagy; as in the fly, autophagy is induced in response to physiological stresses, such as nutrient restriction (Mulakkal et al., 2014), and Atg9 RNAi silencing can reduce this autophagic response (Pircs et al., 2012; Low et al., 2013; Nagy et al., 2013, 2014). Autophagy is also up-regulated during *Drosophila* metamorphosis from larvae to adult-hood (Butterworth et al., 1988; Rusten et al., 2004; Lindmo et al., 2006; Denton et al., 2009, 2013) and autophagosomes increase in abundance in the fat body tissue as the larvae approach puparation (Rusten et al., 2004; Lindmo et al., 2006); enabling the investigation of autophagy under natural conditions without an exogenous stimulus. Here we have used the large size of *Drosophila* fat body cells and organelles, and the capacity for genetic manipulation in the fly, to further investigate the role of Atg9 in autophagy. In this model we observed intraluminal vesicles in Atg8-GFP amphisomes/autolysosomes, which co-located with the endosome marker Rab11 and lysosome marker Lamp1. Upon Atg9 depletion these intraluminal vesicles were no longer detected, suggesting that Atg9 has a specific role in intraluminal vesicle formation in autophagic compartments.

## RESULTS

### Atg9 depletion reduced the number and size of autophagosomes at a time point in *Drosophila* development when autophagy is normally up-regulated

**Fig. 1.**
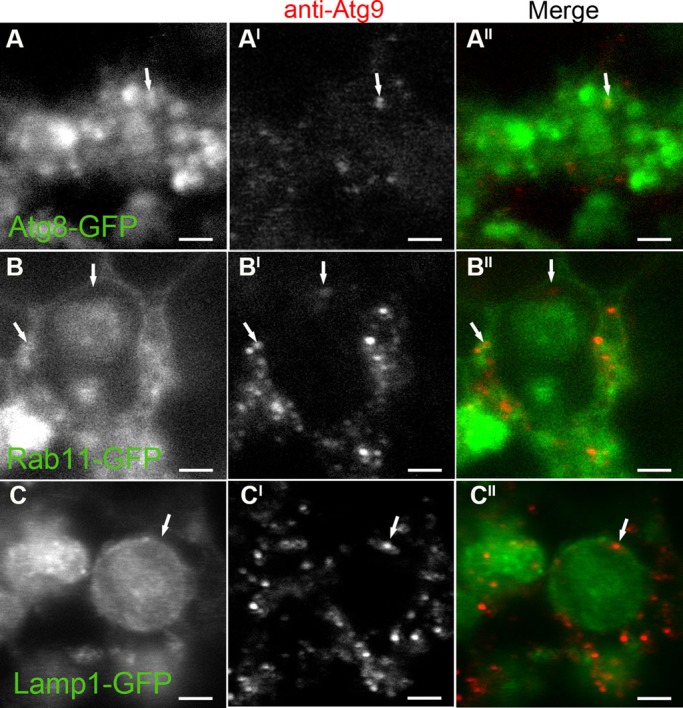
**Cellular localisation of Atg9 in *Drosophila* fat body during development.** Confocal micrographs showing the localisation of Atg9 at 0 h PF, detected with an anti-Atg9 antibody (greyscale in A^I^, B^I^ and C^I^; red in A^II^,B^II^ and C^II^), in relation to Atg8a-GFP (A), Rab11-GFP (B) and Lamp1-GFP (C). The arrows depict the Atg9-positive signal in close proximity to GFP-positive vesicles (A-C^II^). Scale bars=2 μm.

*Drosophila* Atg9 has previously been investigated in the autophagic response to starvation and hypoxia (Pircs et al., 2012; Low et al., 2013; Tang et al., 2013), but its involvement in developmental autophagy has yet to be defined. Here we investigated Atg9 in relation to either Atg8 (another autophagy marker), Rab11 (an endosomal marker) or Lamp1 (an endosomal-lysosomal marker), in fat body tissue at puparium formation (0 h PF), when autophagy is known to be up-regulated (Rusten et al., 2004; Lindmo et al., 2006). There was an increased amount of the Atg9 protein, detected by western blotting, in wild-type fat body tissue at 0 h PF when compared to −4 h PF (supplementary material Fig. S1A). At 0 h PF, Atg9 co-located with Atg8a-GFP in fat body tissue, but not all Atg8a-GFP compartments were positive for Atg9 (Fig. 1A-A^II^). At this time point Atg9 was also detected in association with large Rab11-GFP compartments that in most cases contained intraluminal Rab11-GFP positive vesicles (Fig. 1B-B^II^). Small Rab11 positive vesicles were also observed in close proximity to larger Rab11-GPF compartments and some of these compartments contained Atg9 (Fig. 1B-B^II^). Atg9 was detected in association with Lamp1-GFP compartments that contained intraluminal Lamp1-GFP positive vesicles (Fig. 1C-C^II^). Atg9 was mainly detected as discrete punctate staining when associated with Atg8, Rab11 and Lamp1 compartments (Fig. 1).

**Fig. 2.**
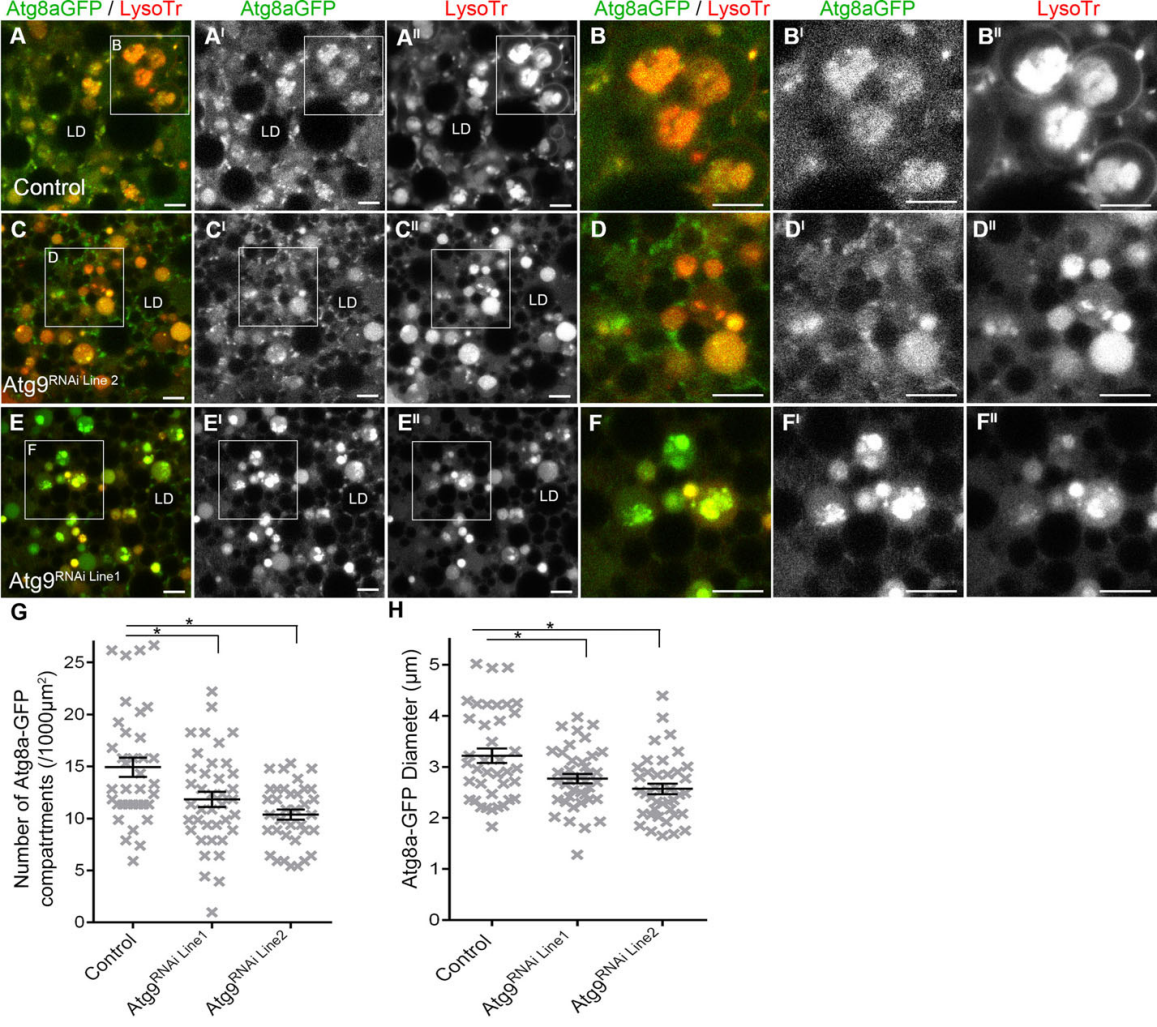
**Depletion of Atg9 by RNAi silencing reduced the number and size of autophagic compartments, but did not impair acidification.** (A-F^II^) Confocal micrograph showing *Drosophila* fat body cells at 0 h PF labelled with Atg8a-GFP (green in A-F; greyscale in A^I^-F^I^) and LysoTracker^®^ (red in A-F; greyscale in A^II^-F^II^). Representative images from; heterozygous CG-GAL4, control (A,B), CG-GAL4>UAS-Atg9^RNAi Line1^ (C,D) and CG-GAL4>UAS-Atg9^RNAi Line2^ (E,F). LD, Lipid droplet; Scale bars=5 μm. (G) Scatter plot showing the number of Atg8a-GFP compartments per 1000 µm^2^. (H) Scatter plot showing the average size of Atg8aGFP/LysoTracker^®^ positive compartments. (G,H) Data is represented as mean±s.e.m. for each genotype; each cross represents quantification from one image. Asterisks indicated significant differences between genotype as calculated by ANOVA with Dunnett post hoc test (*P*<0.05).

To confirm that Atg9 functions in developmental autophagy the formation of Atg8a-GFP autophagosomes was investigated following the depletion of Atg9 by RNAi silencing. Atg9 RNAi silencing, by two independent RNAi lines (BL34901, hereafter referred to as Atg9^RNAi Line1^; and v10045, Atg9^RNAi Line2^) significantly reduced the amount of Atg9 protein detected in fat body tissue by western blotting and *Atg9* mRNA measured by qPCR (*P*<0.05; supplementary material Fig. S1). In control *Drosophila* fat body cells at 0 h PF an average number of 14.9±0.9 Atg8a-GFP positive compartments were detected per 1000 µm^2^ of cell area (visualised in Fig. 2A,A^I^ and quantified in Fig. 2G) and 70±3% of these Atg8a-GFP positive compartments were LysoTracker^®^ positive (visualised in Fig. 2A,A^II^). These Atg8a-GFP/LysoTracker^®^ positive compartments had an average diameter of 3.2±0.1 µm (Fig. 2H), compared to 1.9±0.1 µm for non-LysoTracker^®^ Atg8a-GFP positive compartments. Atg9 depletion significantly reduced the number of Atg8a-GFP compartments in *Drosophila* fat body cells at 0 h PF, when compared to the controls (*P*<0.05; Fig. 2G; 10.4±0.7 and 9.4±0.55 compartments per 1000 µm^2^ respectively for Atg9^RNAi Line1^ and Atg9^RNAi Line2^). While there was a reduction in the number of Atg8a-GFP compartments following Atg9 depletion 61±4% (Atg9^RNAi Line1^) and 78±4% (Atg9^RNAi Line2^) of these autophagosome compartments were still positive for LysoTracker^®^ (i.e. a similar percentage to controls; visualised in Fig. 2C,C^II^,E,E^II^). However, the diameter of these Atg8a-GFP/LysoTracker^®^ compartments was significantly reduced by Atg9 depletion (Fig. 2H; average diameter of 2.8±0.1 µm in Atg9^RNAi Line1^and 2.6±0.1 µm in Atg9^RNAi Line2^; *P*<0.05). The size of Atg8a-GFP only compartments was also significantly reduced in Atg9^RNAi Line2^ (average diameter of 1.7±0.0 µm) when compared to the controls (*P*<0.05), but this was not statistically significant for Atg9^RNAi Line1^ (average diameter of 1.9±0.1 µm).

### Atg9 is required for Rab11 intraluminal vesicles and compartmentalised acidification in amphisomes during developmental autophagy

**Fig. 3.**
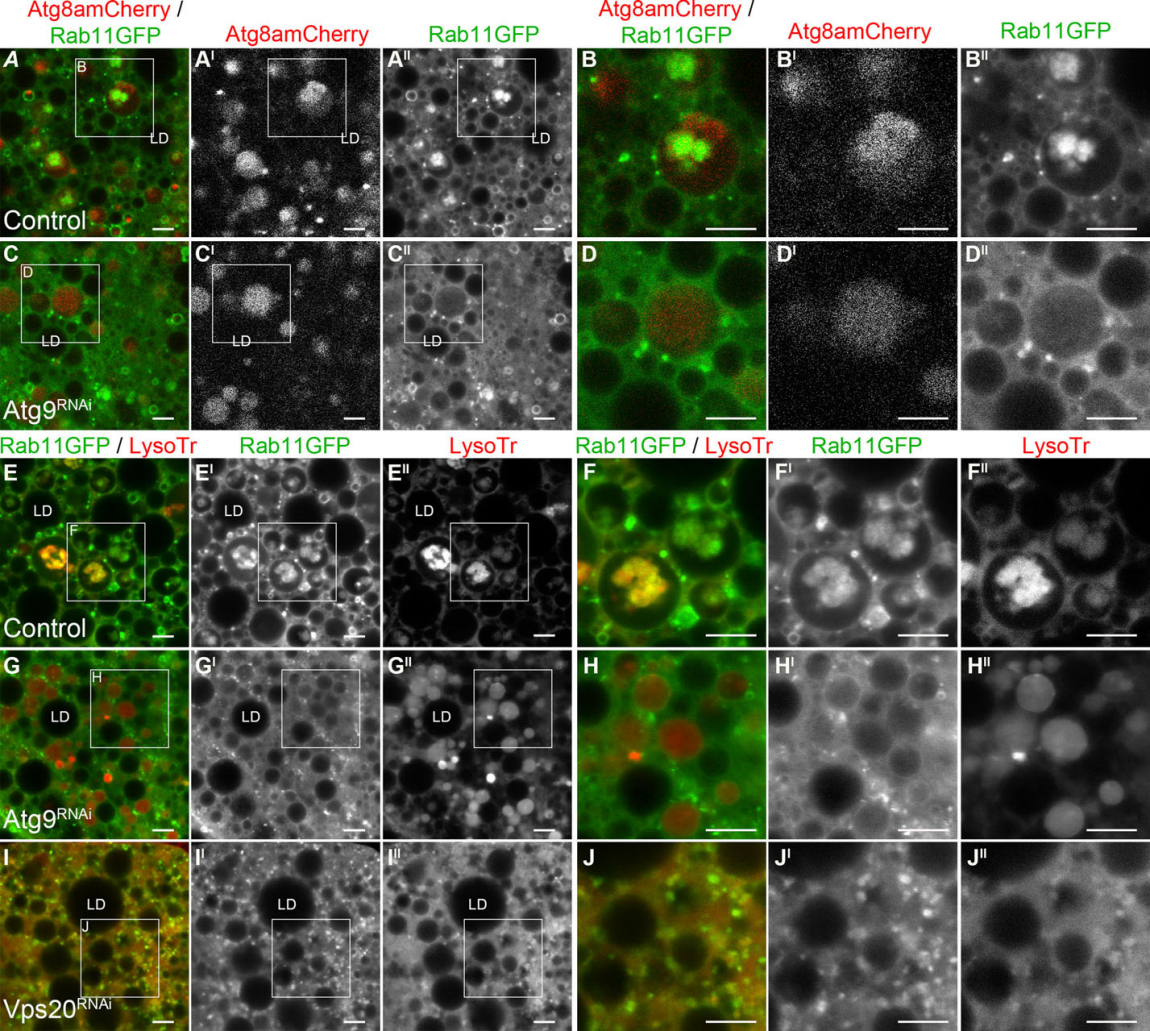
**Intraluminal Rab11 compartments were absent from autophagic compartments in Atg9 depleted fat body cells.** Confocal micrographs of *Drosophila* fat body cells at 0 h PF labelled with Atg8a-mCherry (red in A-D, greyscale in A^I^-D^I^) and Rab11-GFP (green in A-D; greyscale in A^II^-D^II)^; or Rab11-GFP (green in E-J; greyscale in E^I^-J^I^) and LysoTracker^®^ (red in E-J; greyscale in E^II^-J^II^). Representative images from heterozygous CG-GAL4, controls (A,B,E,F), CG-GAL4>UAS-Atg9RNAi Line2 (C,D,G,H) and CG-GAL4>UAS-Vps20RNAi (I,J). LD: Lipid droplet; Scale bars=5 μm.

In control *Drosophila* fat body cells at 0 h PF, Atg8a-mCherry co-located to 75±8% of large Rab11-GFP compartments (>5 μm^2^; Fig. 3A-A^II^), which contained intraluminal Rab11-GFP vesicles (Fig. 3A,A^I^). In Atg9 depleted *Drosophila* fat body cells at 0 h PF, there were large Atg8a-mCherry compartments interacting with Rab11-GFP vesicles (Fig. 3C-C^II^), however, there were no detectable intraluminal Rab11-GFP vesicles in these compartments (Fig. 3C,C^I^). In control *Drosophila* fat body cells, the Rab11-GFP intraluminal vesicles were LysoTracker^®^ positive (co-localised with Rab11; Fig. 3E-E^II^), whereas in Atg9 depleted *Drosophila* fat body cells the entire lumen appeared to be acidified/ LysoTracker^®^ positive (no Rab11 co-localisation; Fig. 3G-G^II^; supplementary material Fig. S2). These LysoTracker compartments were also positive for Rab7 in both controls and Atg9^RNAi^ fat body tissues (supplementary material Fig. S3) Interestingly, at an earlier developmental time point when there is normally minimal developmental autophagy, starvation induced autophagy resulted in similar changes to Rab11 intraluminal compartment morphology and acidification in fat body cells from *Drosophila* with Atg9^RNAi^ silencing (supplementary material Fig. S4); indicating that the changes to Rab11 intraluminal vesicles and acidification were not just the result of a developmental phenomenon. Depletion of the endosomal ESCRT-III protein Vps20 by RNAi silencing also resulted in a loss of Rab11-GFP intraluminal vesicles, but the LysoTracker^®^ was not sequested into compartments and had a cytoplasmic distribution (Fig. 3I-I^II^). As LysoTracker^®^ is a acidotrophic dye this may suggest that the endosomal compartments are not appropriately acidified to cause accumalation of the dye in late endosomes or lysosomes.

### Atg9 depletion reduced the size of autolysosomes and altered the compartmentalisation of LysoTracker^®^ and Lamp1 intraluminal vesicles in fat body cells during developmental autophagy

**Fig. 4.**
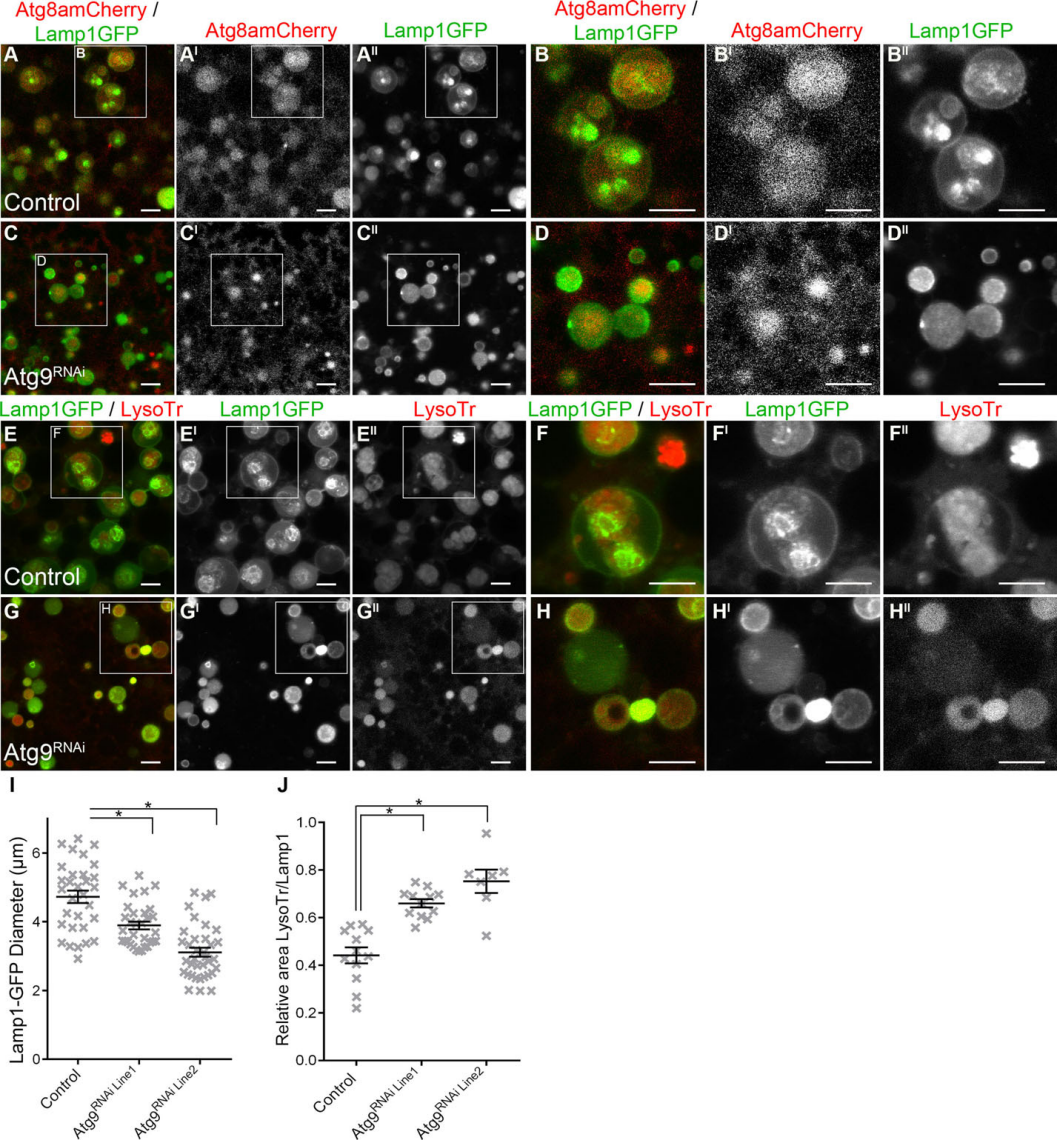
**Lamp1 autophagic compartments had altered intraluminal acidification and a reduced size.** (A-H^II^) Confocal micrograph showing *Drosophila* fat body cells at 0 h PF labelled with (A-D^III^) Atg8a-mCherry (red in A-D; greyscale in A^I^-D^I^) and Lamp1-GFP (green in A-D; greyscale in A^II^-D^II^); or (E-H^III^) Lamp1-GFP (green in E-H; greyscale in E^I^-H^I^) and LysoTracker^®^ (red in E-H; greyscale in E^II^-H^II^). Representative images from heterozygous CG-GAL4, control (A,B,D,E) and CG-GAL4>UAS-Atg9^RNAi Line2^ (C,D,G,H). Scale bars: 5 μm. (I) Scatter plot showing size of Lamp1-GFP positive compartments (presented as a measure of compartment diameter). (J) Scatter plot showing the size of LysoTracker^®^ intraluminal compartments relative to Lamp1-GFP compartment. (I,J) Data is represented as mean±s.e.m. for each genotype; each cross represents quantification from one image. Asterisks indicated significant differences between genotype as calculated by ANOVA with Dunnett post hoc test (*P*<0.05).

In control *Drosophila* fat body cells at 0 h PF, Atg8a-mCherry co-located with Lamp1-GFP compartments and many of these Lamp1-GFP/Atg8a-mCherry positive compartments contained intraluminal Lamp1-GFP vesicles (Fig. 4A-A^II^). Co-location of Atg8a-mCherry with Lamp1-GFP was also detected in Atg9 depleted *Drosophila* fat body cells, but there was a more uniform distribution of Lamp1-GFP in these compartments (Fig. 4C,C^I^,E,E^I^). In addition, in the Atg9 depleted *Drosophila* fat body cells the Lamp1-GFP positive compartments were significantly smaller (*P*<0.05; Fig. 4I; 3.9±0.1 µm in Atg9^RNAi Line1^ and 2.9±0.1 µm in Atg9^RNAi Line2^) than the controls (4.7±0.2 µm). In control *Drosophila* fat body cells LysoTracker^®^ was compartmentalised into intraluminal vesicles within Lamp1-GFP compartments (Fig. 4E-E^II^), while for the Atg9 depleted *Drosophila* fat body cells there was a more uniform distribution of LysoTracker^®^ in the Lamp1-GFP compartments (Fig. 4G-G^I^; supplementary material Fig. S2B). Consequently, the area of the Lamp1-GFP compartments that was acidified in Atg9 depleted *Drosophila* fat body cells was greater than that in the control cells (*P*<0.05; Fig. 4J). In control *Drosophila* fat body cells the intraluminal vesicles within Lamp1-GFP compartments were also positive for intraluminal Rab11 (supplementary material Fig. S4).

### Atg9 depletion abrogated Rab11 intraluminal vesicle formation and impaired midgut degradation in *Drosophila* during metamorphosis

**Fig. 5.**
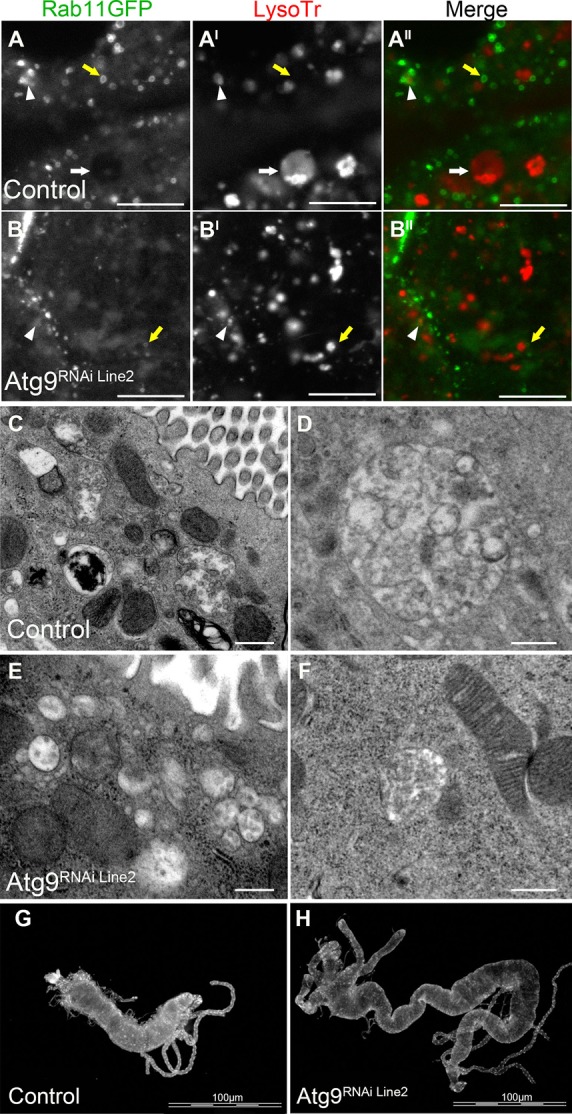
**Atg9 depletion reduced the size of Rab11 and LysoTracker compartments and prevented intraluminal vesicles in the gastric caeca, resulting in reduced gut degradation.** (A-B^II^) Confocal micrograph of *Drosophila* midgut cells at 0 h PF labelled with Rab11-GFP (greyscale in A,B; green in A^II^,B^II^), and LysoTracker^®^ (greyscale in A^I^,B^I^; red in A^II^,B^II^). Arrow heads indicate Rab11-GFP in close proximity to LysoTracker^®^ (A,B), white arrows indicate enlarged LysoTracker^®^ compartments (A), yellow arrows depicted Rab11-GFP as large rings (A,B). Scale bar: 10 μm. (C-F) TEM micrograph of *Drosophila* midgut cells at 0 h APF, illustrating subcellular compartments containing intraluminal vesicles. Scale bar: 500 nm. (G,H) Fluorescence image of Hoechst stained midguts from +4 h PF pupae. Scale bar: 100 μm. Representative images from heterozygous NP1-GAL4, control (A,C,D,G) and NP1-GAL4>UAS-Atg9^RNAi Line2^ (B,E,F,H).

The *Drosophila* larval midgut is degraded by autophagy during metamorphosis (Lee et al., 2002; Denton et al., 2009). In Atg9 depleted *Drosophila* midgut cells at 0 h PF, the Rab11-GFP and LysoTracker^®^ compartments were small and the morphology was altered when compared to control midgut cells (Fig. 5A-B^II^). This difference in compartment morphology was particularly evident for LysoTracker^®^, with control midgut cells having larger compartments that contained intraluminal vesicles (Fig. 5A^I^,A^II^) and Atg9 depleted midgut cells having smaller compartments with little evidence of compartmentalisation (Fig. 5B^I^,B^II^). TEM analysis of *Drosophila* midgut cells at 0 h PF supported this altered compartment morphology, with control midgut cells having larger vesicles with multiple intraluminal vesicles (Fig. 5C,D), whereas the compartments in Atg9 depleted midgut cells had either a uniform granular appearance or only limited evidence of intraluminal vesicles (Fig. 5E,F). Consequently, there were significantly more multivesicular structures (*P*<0.03) identified in control midgut cells (12.2±1.9) than Atg9^RNAi Line2^ midgut cells (6.9±1.2). This appeared to affect autophagic degradation as at +4 h PF the control *Drosophila* pupae had shorter midguts with an average perimeter of 2273±57 µm and gastric caeca that were almost completely degraded (Fig. 5G), whereas the midguts from Atg9^RNAi Line2^ pupae were significantly longer than controls (*P*<0.05) with an average perimeter of 6363±368 µm and gastric caeca that remained intact (Fig. 5H).

## DISCUSSION

Autophagy is a cellular degradation and recycling process that is important during energy metabolism, lipid metabolism, physiological stress, tissue remodelling and organism development (Singh and Cuervo, 2011). Changes in autophagy progression have been shown to be important in numerous human pathologies including neurological disorders, cancer and infectious diseases (Choi et al., 2013). Defining the mechanism and regulation of autophagy is essential to fully understand the pathophysiology of these diseases and this may lead to the design of new targeted therapies. Atg9 is the only transmembrane protein known to be involved in autophagy and it is thought to function in early autophagosome formation, however, it localises to multiple cellular locations (Young et al., 2006; Orsi et al., 2012; Puri et al., 2013) and is not thought to be integrated into autophagosome membranes (Orsi et al., 2012). We have demonstrated that Atg9 has a function relating to intraluminal vesicle formation and is required for compartmentalised acidification within amphisomes and autolysosomes.

In yeast, Atg9 is required for autophagy and its depletion prevents the formation of autophagosomes that are induced by rapamycin treatment or through nitrogen/amino acid starvation (Suzuki et al., 2001). In mammals, the depletion of Atg9 also reduces autophagic activity, limiting LC3-I to LC3-II lipidation, reducing the number of early autophagic structures or LC3 puncta formed, and decreasing the turnover of long lived proteins (Yamada et al., 2005; Young et al., 2006; Saitoh et al., 2009; Orsi et al., 2012). Similarly, in *Drosophila*, the depletion of Atg9 by RNAi silencing reduced the number of autophagic structures detected in fat body cells following starvation of third instar larvae and also reduced protein clearance (Low et al., 2013; Tang et al., 2013; Nagy et al., 2014). The reduction in both the number and the size of autophagosomes during development, which were observed upon RNAi silencing of *Atg9* in the fat body, would be compatible with previous reports for roles of Atg9 in membrane recruitment to the phagophore and for membrane expansion (Suzuki et al., 2001; Yamada et al., 2005; Young et al., 2006; Saitoh et al., 2009; Orsi et al., 2012; Lamb et al., 2013b; Low et al., 2013; Tang et al., 2013; Nagy et al., 2014). Although there was reduced formation of autophagosomes in *Atg9* RNAi silenced fat body tissues during development, autophagic compartments were still formed allowing us to track autophagosome maturation in Atg9 depleted tissue.

Autophagosomes interact with endosomes and lysosomes in a maturation process that sequentially generates amphisomes and then autolysosomes. This autophagosome maturation process is essential for the delivery of endolytic and exolytic acid hydrolases, as well as the generation of the acidic conditions, which enable the degradation of the luminal content of autophagosomes (Gordon and Seglen, 1988; Dunn, 1990; Fengsrud et al., 1995). In mammalian cells, Atg9 has been observed to be trafficked via the endosomal network and under normal nutrient conditions is often associated with recycling endosomes (Longatti et al., 2012; Orsi et al., 2012; Puri et al., 2013). The induction of autophagy by starvation decreases the association of Atg9 with recycling endosomes (Longatti et al., 2012; Orsi et al., 2012), but increases Atg9 localisation with late endosomes (Young et al., 2006; Tang et al., 2011). We also observed Atg9 localised to compartments that were positive for the endosome and lysosome markers Rab11 and Lamp1 in *Drosophila* fat body, at a developmental time point when autophagy is induced by hormone signalling. These observations have led to the hypothesis that Atg9 might have a role in autophagosome maturation (Takahashi et al., 2011; Reggiori and Tooze, 2012). We observed, however, Atg8 autophagosome acidification and co-location of Rab7, Rab11 and Lamp1 with these compartments following Atg9 depletion, indicating that amphisome and autolysosome formation was not abrogated. This implied that the recruitment of endosomes and lysosomes, and the vesicular fusion machinery that mediates the fusion of these degradative organelles to form respectively amphisomes and autolysosomes, was not impaired by Atg9 depletion. Despite the capacity to form amphisomes and autolysosomes, there appeared to be a disruption to developmental autophagy and the reduced turnover of midgut tissue, suggesting that the degradative properties of these compartments might still be impaired.

**Fig. 6.**
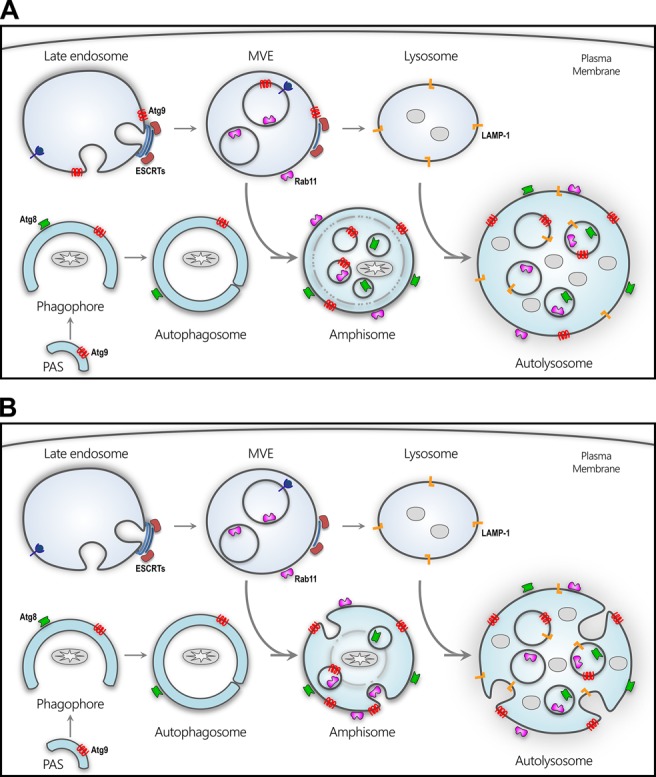
**Potential role of Atg9 in autophagosome maturation.** Schematic showing the autophagic progression from initiation at the PAS, membrane elongation to form the phagophore, membrane closure to encapsulate a mitochondrion in the autophagosome which then fuses with a multivesicular endosome to form an amphisome and finally fusion with a lysosome to produce an autolysosome. (A) Atg9 potentially facilitates the formation of intraluminal vesicle at the late endosome to form a multivesicular endosome prior to fusion with an autophagosome. (B) Atg9 also potentially facilitates intraluminal vesicle formation in amphisomes and autolysosome. PAS, phagophore assembly site; Atg, autophagy related gene.

Impaired autophagic degradation can result from abrogated fusion with endosome and lysosome compartments, causing either the accumulation of autophagosomes, (Eskelinen, 2006; Lee et al., 2007; Fader and Colombo, 2009; Fader et al., 2009) or the enlargement of autolysosomes (Settembre et al., 2008a,b). Atg9 depletion resulted in smaller amphisomes/autolysosomes and no apparent accumulation of autophagosomes, further indicating that amphisomes and autolysosomes are forming in these tissues. In Atg9 depleted tissues there were, however, significant morphological changes to amphisomes-autolysosomes, with the loss of intraluminal vesicles, which were detectable by Rab7, Rab11, Lamp1 or LysoTracker^®^ and a loss of intraluminal vesicles was also noted by TEM of *Drosophila* midgut tissue. In late endosomes, intraluminal vesicles are formed to allow the internalisation of ubiquitinated proteins, membrane rafts, other specific lipid domains and membrane receptors/cargo (Babst, 2011). Amphisomes result from the fusion of autophagosomes with these multivesicular endosomes (Tamai et al., 2007; Fader et al., 2008, 2009) and Rab11 multivesicular structures increase in size and co-localise with autophagosomal markers following the induction of autophagy (Fader et al., 2008). Atg9 localises with a number of endosomal markers, which are associated with late endosomes and multivesicular endosomes, including Rab11 and Rab7 (Denzer et al., 2000; Satoh et al., 2005; Savina et al., 2005; Fader et al., 2008; Vanlandingham and Ceresa, 2009); and clusters of Atg9 positive tubules/vesicles have been shown to emanate from these multivesicular endosomes (Orsi et al., 2012). This could suggest that Atg9 functions in intraluminal vesicle formation in endosomes, prior to amphisome formation (Fig. 6A). The ECSRT machinery that is required for intraluminal vesicle formation in endosome maturation is also required for autophagosome maturation; as RNAi depletion or mutations in the ECSRT machinery can cause the accumulation of autophagosomes (Filimonenko et al., 2007; Lee et al., 2007; Rusten et al., 2007; Manil-Segalen et al., 2012). Here, RNAi depletion of the ESCRT-III subunit Vps20, prevented the generation of acidified endosomes and Rab11 amphisomes and autolysosomes, instead generating Rab11 compartments similar to class E-compartments (Doyotte et al., 2005). Paradoxically, while Atg9 RNAi affected intraluminal vesicle formation it did not impair the formation of amphisomes and autolysosomes, suggesting that Atg9 does not affect endosome maturation to the same extent as the depletion of the ESCRT machinery.

The intraluminal vesicles observed in amphisomes and autolysosomes, were positive for Lamp1 and Atg8, proteins thought to associate respectively with the membranes of lysosomes and autophagosomes; in addition to Rab11 which may also be present on multivesicular endosomes. Though Lamp1 protein has been detected on both endosomes and lysosomes it is enriched in lysosomal compartments and consequently preferentially associated with autolysosomes rather than amphisomes (Schroder et al., 2010). In *Drosophila* wing discs, Lamp1 has also been detected on the intraluminal vesicles in multivesicular compartments (Rusten et al., 2006) and in larval fat body Lamp1 intraluminal vesicles have been observed within Lamp1/Atg8 positive compartments following starvation (Kim et al., 2012). These intraluminal vesicles observed in amphisomes-autolysosomes could be formed by the fusion of autophagosomes with multivesicular endosomes (Bright et al., 2005; Yu et al., 2010; Chen and Yu, 2013), but from our data we could not exclude direct formation of intraluminal vesicles by amphisomes or autolysosomes (Fig. 6). Intraluminal vesicle formation in the autophagosome may facilitate cargo sorting, in a manner similar to late endosomes. This cargo sorting may be necessary for autophagosomes, as not all proteins targeted by autophagy are destined for lysosomal degradation, and autophagy can also act as a trafficking pathway for proteins such as, interleukins and lipoproteins (Pan et al., 2008; Deretic et al., 2012; Ouimet, 2013). Intraluminal vesicles may also be important in establishing local pH gradients, as variations in intraluminal acidity were detected by LysoTracker^®^ following Atg9 depletion.

It is speculated that the localised acidification observed in amphisomes-autolysosomes may also be used to facilitate the degradation of specific cargo incorporated into autophagosomes. The hydrolytic enzymes delivered by endosomes and lysosomes to autophagosomes require an acidic environment to function effectively (Mindell, 2012) and increased lysosome pH is known to decrease protein degradation in autolysosomes (Mousavi et al., 2001; Zhang et al., 2013; Hosogi et al., 2014). The low pH environment of endosomes, lysosomes and autolysosomes is controlled by vesicular H^+^ATPase complexes, which act as a proton pump to facilitate acidification (Forgac, 2007). We observed compartmentalisation of LysoTracker^®^ in control, but not Atg9 depleted cells which may explain the reduced degradative capacity and inability to degrade midgut tissue when Atg9 was depleted in this tissue. Inability to distribute the H^+^ATPase complex to the lumen of degradative vesicles might be central to this impaired degradative capacity (Shen and Mizushima, 2014) and be required to generate a localised low pH environment.

Atg9 appears to have an important functional role in intraluminal vesicle formation and it is clear that this compartmentalisation is required to develop an appropriately localised acidic environment within degradative compartments. While Atg9 has a role in intraluminal vesicle formation in endosomes, it remains to be established whether these intraluminal vesicles can be formed directly in amphisomes and autolysosomes (Fig. 6). Previous studies have implied that Atg9 has a role in autophagosome maturation, but from this study it was evident that this role might not involve vesicular fusion with endosomes and lysosomes as previously suggested. Rather, Atg9 may function in the formation of intraluminal vesicles that are required for correct amphisome and autolysosome function. Atg9 could facilitate the recruitment of the molecular machinery that is required for intraluminal vesicular formation either at the late endosome to form multivesicular endosomes or at the amphisome-autolysosome following endosome fusion. During autophagy initiation, Atg9 has been shown to be involved in the recruitment of a range of molecular machinery including tethering proteins, Rab trafficking proteins and autophagy proteins (Kakuta et al., 2012; Wang et al., 2013; Papinski and Kraft, 2014; Papinski et al., 2014), and it may play a similar role at the late endosome or amphisome-autolysosome. This effectively adds to the potential role of Atg9 in intracellular trafficking (Saitoh et al., 2009; Popovic and Dikic, 2014), the induction of autophagy and PAS coordination (Orsi et al., 2012), as well as the purported roles in vesicular interaction and vesicle fusion (Webber and Tooze, 2010). It remains to be established how Atg9 facilitates intraluminal vesicle formation and compartmentalised acidification.

## MATERIALS AND METHODS

### Drosophila stocks

*Drosophila* stocks were maintained in standard medium at 25°C, with a 12:12 h light to dark schedule. The yeast *GAL4-UAS* system was used for targeted gene expression (Brand and Perrimon, 1993). Fat body specific expression of transgenes from the UAS was driven by *CG-GAL4* (Asha et al., 2003) and gut specific expression of transgenes from the UAS was driven by *NP1-GAL4* (*Drosophila* genetic resource center, Kyoto). Wild-type and transgenic stock *UAS-Atg9^RNAi^* (stock #34901) were obtained from the Bloomington *Drosophila* Stock Center (Indiana University, Bloomington, USA). RNAi silencing stocks *UAS-Atg9^RNAi^* (stock #v10045) and *UAS-Vps20^RNAi^* (stock #v26388) were obtained from the Vienna *Drosophila* RNAi Centre (Vienna, Austria). *UAS-Lamp1-GFP* was obtained from Helmut Kramer (Center for Basic Neuroscience, Dallas, USA; Pulipparacharuvil et al., 2005). The autophagy markers *Atg8a-GFP* and *Atg8a-Cherry* were driven by an endogenous promoter and kindly provided prior to publication by Erik Baehrecke (University of Massachusetts, Medical School, MA, USA). *UAS-Rab11-GFP* was obtained from Markos González-Gaitán (University of Geneva, Geneva, Switzerland; Entchev et al., 2000; Wucherpfennig et al., 2003) and Donald F. Ready (Purdue University, West Lafayette, USA; Satoh et al., 2005).

### Gene expression and protein analysis

For quantitative real-time PCR (qRT-PCR) analysis, RNA was isolated from the fat body tissue of 20 larvae, using an RNAqueous^®^ kit (Ambion, Auston, USA) according to the manufacturer's protocol. cDNA was synthesized using a High Capacity RNA-to-cDNA kit (Applied Biosystems, Waltham, USA). Quantitative RT-PCR was performed using a 7500 Fast Real-Time PCR System using a Fast SYBR Green Master Mix kit (Applied Biosystems). PCR primers were obtained from GeneWorks (Adelaide, Australia). The following primers were used to assay the *in vivo* efficiency of *UAS-RNAi* transgenes: *Atg9* (CG 3615) forward, 5′-AGC AGA AGC ACG GAT TCA CA-3′, and reverse, 5′-GCA GTG CAT CAC AAA GGC AA-3′ and *rp49* (CG7939, used as an endogenous control) forward, 5′-CGAGTTGAACTGCCTTCAAGATGACCA-3′, reverse 5′-GCTTGGTGCGCTTCTTCACGATCT-3′. Three independent biological samples were analysed for each genotype, and the mRNA expression levels were normalized against the endogenous control gene *rp49*, using the ΔΔC_T_ method.

For western blotting, the fat body tissue was extracted from 10–15 either late 3rd larval instars [−4 h from puparium formation (−4 h PF)] or newly formed white pupae (0 h PF) using a previously described method (Shandala et al., 2011). Larval development was determined according to the method described by Andres and Thummel (1994). For immunoblotting, protein was separated by SDS-PAGE (30 μg protein load) and then transferred to nitrocellulose membranes (Shandala et al., 2011). The membranes were probed with either rabbit anti-Atg9 antibody at 1/200 (Novus Biologicals, Littleton, USA) or for a loading control goat anti-GADH at 1/1000 (Imgenex, Littleton USA) primary antibodies and a horseradish-posixidate conjugate secondary antibody. Proteins were visualised using Novex^®^ electro chemiluminescent substrate reagent kit (Life Technology, Carlsbad, USA) and imaged using a ImageQuant™ LAS 4000 imager, software version 1.2.0.101 (GE Healthcare Bioscience, Parramatta, Australia). Quantification was performed using AlphaViewSA™ software version 3.0 (ProteinSimple, Santa Clara, USA).

### Immunostaining and microscopy

For immunostaining, *Drosophila* fat body tissue was mounted on a microscope slide and fixed in 2% (v/v) paraformaldehyde in PBS, for one hour on ice. The slides were then washed in 0.1% (v/v) Tween 20 (Sigma-Aldrich, St Louis, USA) in PBS for 30 min. Non-specific interactions were blocked by incubating the tissues with 5% (v/v) BSA (Sigma-Aldrich) and 0.1% (v/v) Tween 20 in PBS for 30 min, before incubation with rabbit anti-Atg9 antibody (1/50; Novus Biologicals, Littleton, USA) or goat anti-Rab11 antibodies (1/100; obtained from Robert S. Cohen, University of Kansas; (Dollar et al., 2002), diluted in 0.1% (v/v) Tween 20 and 5% (v/v) BSA in PBS for two hours at RT and then overnight at 4°C. The tissues/slides were washed in 0.1% (v/v) Tween in PBS for 40 min and then incubated with either goat anti-rabbit IgG or donkey anti-goat IgG, secondary Cy5 labelled antibodies (Jackson ImmunoResearch Laboratories, West Grove, USA), for two hours at RT. The slides were washed in 0.1% (v/v) Tween 20 in PBS for 40 min and then mounted in 80% (v/v) glycerol in PBS. For live cell imaging *Drosophila* fat body and gut tissues were stained with LysoTracker^®^ Red (1/200; Life Technology) for two minutes, then mounted in carbomer-940 (Snowdrift Farm, Tucson, USA) based optical coupling gel (Rothstein et al., 2006). Imaging was performed using a Zeiss LSM710 NLO confocal microscope equipped with Argon-gas and 543 nm and 633 nm solid-state lasers (Zeiss, Oberkochen, Germany) and a two-photon Mai-Tai^®^, tuneable Ti:Sapphire femtosecond pulse laser (Spectra-Physics, St Clara, USA). Images were captured using a 63× oil immersion lens. Each confocal micrograph represented 1.5 μm thin optical sections.

For gut length analysis, the midguts were dissected from +4 h PF larvae into PBS and fixed in 4% (v/v) paraformaldehyde (Denton et al., 2009). Tissues were stained with Hoechst 33258 (Invitrogen, USA) and imaged with an Olympus IX71 epifluorescence microscope (Olympus, Japan). Tissues for transmission electron microscopy (TEM) analysis were prepared as described previously (Kohler et al., 2009) and viewed with an FEI Tecnai G2 Spirit TEM (FEI, Hillsboro, USA).

### Image analysis

Analysis of compartment number and size was performed using Volocity^®^ 3D Imaging Software (PerkinElmer, Waltham, USA). For each genotype and marker combination, micrographs from a minimum of 10 biological replicates were each analysed over an area of 2025 μm^2^. For the analysis of the number and size of Atg8a-GFP compartments an object finder function was used, allowing automated detection based on compartment intensity at a specific threshold. Lamp1-GFP compartments were measured manually as the sub-compartmentalisation did not allow for automated detection. To assess the changes in Lamp1-GFP intraluminal compartments, a region of interest or ROI was drawn around the Lamp1-GFP compartment, and object finder was used to measure the area of the intraluminal compartments filled with LysoTracker^®^. An average number of 13 Lamp1-GFP compartments were measured per micrograph (range 8-38). The data was presented as a mean±s.e.m, and the treatment groups compared by ANOVA analysis in GraphPad Prism with Dunnett post-hoc analysis (Prism software, version 6.01, USA).

The length around the perimeter of the midgut (from the foregut/midgut to midgut/foregut junction) was used to define midgut length and analysed using AnalySIS Software (Olympus, Shinjuku, Japan). At least 12 midguts from each genotype were examined. The data was presented as a mean±s.e.m. and the different genotypes compared by a Student's *t*-test using GraphPad Prism. The number of multivesicular structures (intracellular compartments that contained smaller intraluminal vesicles) in each midgut was determined manually from a total of 13 TEM images, from three independent biological replicates for each genotype, and involved a capture field view of 8.5 μm×8.5 μm, at 11,500× magnification.

## References

[BIO013979C1] Andres A. J. and Thummel C. S. (1994). Methods for quantitative analysis of transcription in larvae and prepupae. *Methods Cell Biol.* 44, 565-573. 10.1016/S0091-679X(08)60932-2 7535884

[BIO013979C2] Asha H., Nagy I., Kovacs G., Stetson D., Ando I. and Dearolf C. R. (2003). Analysis of Ras-induced overproliferation in Drosophila hemocytes. *Genetics* 163, 203-215. 12586708 10.1093/genetics/163.1.203 PMC1462399

[BIO013979C3] Babst M. (2011). MVB vesicle formation: ESCRT-dependent, ESCRT-independent and everything in between. *Curr. Opin. Cell Biol.* 23, 452-457. 10.1016/j.ceb.2011.04.008 21570275 PMC3148405

[BIO013979C4] Berg T. O., Fengsrud M., Strømhaug P. E., Berg T. and Seglen P. O. (1998). Isolation and characterization of rat liver amphisomes: evidence for fusion of autophagosomes with both early and late endosomes. *J. Biol. Chem.* 273, 21883-21892. 10.1074/jbc.273.34.21883 9705327

[BIO013979C5] Brand A. H. and Perrimon N. (1993). Targeted gene expression as a means of altering cell fates and generating dominant phenotypes. *Development* 118, 401-415. 8223268 10.1242/dev.118.2.401

[BIO013979C6] Bright N. A., Gratian M. J. and Luzio J. P. (2005). Endocytic delivery to lysosomes mediated by concurrent fusion and kissing events in living cells. *Curr. Biol.* 15, 360-365. 10.1016/j.cub.2005.01.049 15723798

[BIO013979C7] Butterworth F. M., Emerson L. and Rasch E. M. (1988). Maturation and degeneration of the fat body in the Drosophila larva and pupa as revealed by morphometric analysis. *Tissue Cell* 20, 255-268. 10.1016/0040-8166(88)90047-X 3136556

[BIO013979C95] Chen D., Fan W., Lu Y., Ding X., Chen S. and Zhong Q. (2012). A mammalian autophagosome maturation mechanism mediated by TECPR1 and the Atg12-Atg5 conjugate. *Molecular Cell* 45, 629-641 10.1016/j.molcel.2011.12.036 22342342 PMC3299828

[BIO013979C8] Chen Y. and Yu L. (2013). Autophagic lysosome reformation. *Exp. Cell Res.* 319, 142-146. 10.1016/j.yexcr.2012.09.004 22999865

[BIO013979C9] Choi A. M. K., Ryter S. W. and Levine B. (2013). Autophagy in human health and disease. *N. Engl. J. Med.* 368, 651-662. 10.1056/NEJMra1205406 23406030

[BIO013979C10] Denton D., Shravage B., Simin R., Mills K., Berry D. L., Baehrecke E. H. and Kumar S. (2009). Autophagy, not apoptosis, is essential for midgut cell death in Drosophila. *Curr. Biol.* 19, 1741-1746. 10.1016/j.cub.2009.08.042 19818615 PMC2783269

[BIO013979C11] Denton D., Aung-Htut M. T., Lorensuhewa N., Nicolson S., Zhu W., Mills K., Cakouros D., Bergmann A. and Kumar S. (2013). UTX coordinates steroid hormone-mediated autophagy and cell death. *Nat. Commun.* 4, 2916 10.1038/ncomms3916 24336022 PMC3973156

[BIO013979C12] Denzer K., Kleijmeer M. J., Heijnen H. F., Stoorvogel W. and Geuze H. J. (2000). Exosome: from internal vesicle of the multivesicular body to intercellular signaling device. *J. Cell Sci.* 113, 3365-3374. 10984428 10.1242/jcs.113.19.3365

[BIO013979C13] Deretic V. (2009). Links between autophagy, innate immunity, inflammation and Crohn's disease. *Dig. Dis.* 27, 246-251. 10.1159/000228557 19786748 PMC2788938

[BIO013979C96] Deretic V., Jiang S. and Dupont N. (2012). Autophagy intersections with conventional and unconventional secretion in tissue development, remodeling and inflammation. *Trends Cell Biol.* 22, 397-406. 10.1016/j.tcb.2012.04.008 22677446 PMC3408825

[BIO013979C14] Dollar G., Struckhoff E., Michaud J. and Cohen R. S. (2002). Rab11 polarization of the Drosophila oocyte: a novel link between membrane trafficking, microtubule organization, and oskar mRNA localization and translation. *Development* 129, 517-526. 11807042 10.1242/dev.129.2.517

[BIO013979C15] Doyotte A., Russell M. R. G., Hopkins C. R. and Woodman P. G. (2005). Depletion of TSG101 forms a mammalian “Class E” compartment: a multicisternal early endosome with multiple sorting defects. *J. Cell Sci.* 118, 3003-3017. 10.1242/jcs.02421 16014378

[BIO013979C16] Dunn W. A. (1990). Studies on the mechanisms of autophagy: maturation of the autophagic vacuole. *J. Cell Biol.* 110, 1935-1945. 10.1083/jcb.110.6.1935 2161853 PMC2116125

[BIO013979C97] Entchev E. V., Schwabedissen A. and Gonzalez-Gaitan M. (2000). Gradient formation of the TGF-beta homolog Dpp. *Cell* 103, 981-991. 10.1016/S0092-8674(00)00200-2 11136982

[BIO013979C17] Eskelinen E.-L. (2006). Roles of LAMP-1 and LAMP-2 in lysosome biogenesis and autophagy. *Mol. Aspects Med.* 27, 495-502. 10.1016/j.mam.2006.08.005 16973206

[BIO013979C18] Fader C. M. and Colombo M. I. (2009). Autophagy and multivesicular bodies: two closely related partners. *Cell Death Differ.* 16, 70-78. 10.1038/cdd.2008.168 19008921

[BIO013979C19] Fader C. M., Sanchez D., Furlan M. and Colombo M. I. (2008). Induction of autophagy promotes fusion of multivesicular bodies with autophagic vacuoles in k562 cells. *Traffic* 9, 230-250. 10.1111/j.1600-0854.2007.00677.x 17999726

[BIO013979C20] Fader C. M., Sanchez D. G., Mestre M. B. and Colombo M. I. (2009). TI-VAMP/VAMP7 and VAMP3/cellubrevin: two v-SNARE proteins involved in specific steps of the autophagy/multivesicular body pathways. *Biochim. Biophys. Acta* 1793, 1901-1916. 10.1016/j.bbamcr.2009.09.011 19781582

[BIO013979C21] Fengsrud M., Roos N., Berg T., Liou W., Slot J. W. and Seglen P. O. (1995). Ultrastructural and immunocytochemical characterization of autophagic vacuoles in isolated hepatocytes: effects of vinblastine and asparagine on vacuole distributions. *Exp. Cell Res.* 221, 504-519. 10.1006/excr.1995.1402 7493651

[BIO013979C22] Filimonenko M., Stuffers S., Raiborg C., Yamamoto A., Malerød L., Fisher E. M. C., Isaacs A., Brech A., Stenmark H. and Simonsen A. (2007). Functional multivesicular bodies are required for autophagic clearance of protein aggregates associated with neurodegenerative disease. *J. Cell Biol.* 179, 485-500. 10.1083/jcb.200702115 17984323 PMC2064794

[BIO013979C23] Forgac M. (2007). Vacuolar ATPases: rotary proton pumps in physiology and pathophysiology. *Nat. Rev. Mol. Cell Biol.* 8, 917-929. 10.1038/nrm2272 17912264

[BIO013979C24] Gordon P. B. and Seglen P. O. (1988). Prelysosomal convergence of autophagic and endocytic pathways. *Biochem. Biophys. Res. Commun.* 151, 40-47. 10.1016/0006-291X(88)90556-6 3126737

[BIO013979C25] Harding T. M., Morano K. A., Scott S. V. and Klionsky D. J. (1995). Isolation and characterization of yeast mutants in the cytoplasm to vacuole protein targeting pathway. *J. Cell Biol.* 131, 591-602. 10.1083/jcb.131.3.591 7593182 PMC2120622

[BIO013979C26] Hosogi S., Kusuzaki K., Inui T., Wang X. and Marunaka Y. (2014). Cytosolic chloride ion is a key factor in lysosomal acidification and function of autophagy in human gastric cancer cell. *J. Cell. Mol. Med.* 18, 1124-1133. 10.1111/jcmm.12257 24725767 PMC4508152

[BIO013979C27] Itakura E., Kishi-Itakura C. and Mizushima N. (2012). The hairpin-type tail-anchored SNARE syntaxin 17 targets to autophagosomes for fusion with endosomes/lysosomes. *Cell* 151, 1256-1269. 10.1016/j.cell.2012.11.001 23217709

[BIO013979C98] Kakuta S., Yamamoto H., Negishi L., Kondo-Kakuta C., Hayashi N. and Ohsumi Y. (2012). Atg9 vesicles recruit vesicle-tethering proteins Trs85 and Ypt1 to the autophagosome formation site. *J. Biol. Chem.* 287, 44261-44269. 10.1074/jbc.M112.411454 23129774 PMC3531741

[BIO013979C28] Kim S., Naylor S. A. and DiAntonio A. (2012). Drosophila Golgi membrane protein Ema promotes autophagosomal growth and function. *Proc. Natl. Acad. Sci. USA* 109, E1072-E1081. 10.1073/pnas.1120320109 22493244 PMC3344964

[BIO013979C29] Klionsky D. J. and Schulman B. A. (2014). Dynamic regulation of macroautophagy by distinctive ubiquitin-like proteins. *Nat. Struct. Mol. Biol.* 21, 336-345. 10.1038/nsmb.2787 24699082 PMC4036234

[BIO013979C99] Kohler K., Brunner E., Guan X. L., Boucke K., Greber U. F., Mohanty S., Barth J. M., Wenk M. R. and Hafen E. (2009). A combined proteomic and genetic analysis identifies a role for the lipid desaturase Desat1 in starvation-induced autophagy in Drosophila. *Autophagy* 5, 980-990. 10.4161/auto.5.7.9325 19587536

[BIO013979C30] Kroemer G., Marino G. and Levine B. (2010). Autophagy and the integrated stress response. *Mol. Cell* 40, 280-293. 10.1016/j.molcel.2010.09.023 20965422 PMC3127250

[BIO013979C31] Kundu M. and Thompson C. B. (2008). Autophagy: basic principles and relevance to disease. *Annu. Rev. Pathol.* 3, 427-455. 10.1146/annurev.pathmechdis.2.010506.091842 18039129

[BIO013979C32] Lamb C. A., Dooley H. C. and Tooze S. A. (2013a). Endocytosis and autophagy: shared machinery for degradation. *Bioessays* 35, 34-45. 10.1002/bies.201200130 23147242

[BIO013979C33] Lamb C. A., Yoshimori T. and Tooze S. A. (2013b). The autophagosome: origins unknown, biogenesis complex. *Nat. Rev. Mol. Cell Biol.* 14, 759-774. 10.1038/nrm3696 24201109

[BIO013979C34] Lee C.-Y., Cooksey B. A. K. and Baehrecke E. H. (2002). Steroid regulation of midgut cell death during Drosophila development. *Dev. Biol.* 250, 101-111. 10.1006/dbio.2002.0784 12297099

[BIO013979C35] Lee J.-A., Beigneux A., Ahmad S. T., Young S. G. and Gao F.-B. (2007). ESCRT-III dysfunction causes autophagosome accumulation and neurodegeneration. *Curr. Biol.* 17, 1561-1567. 10.1016/j.cub.2007.07.029 17683935

[BIO013979C36] Lindmo K., Simonsen A., Brech A., Finley K., Rusten T. E. and Stenmark H. (2006). A dual function for Deep orange in programmed autophagy in the Drosophila melanogaster fat body. *Exp. Cell Res.* 312, 2018-2027. 10.1016/j.yexcr.2006.03.002 16600212

[BIO013979C37] Liu E. Y. and Ryan K. M. (2012). Autophagy and cancer--issues we need to digest. *J. Cell Sci.* 125, 2349-2358. 10.1242/jcs.093708 22641689

[BIO013979C38] Longatti A., Lamb C. A., Razi M., Yoshimura S.-I., Barr F. A. and Tooze S. A. (2012). TBC1D14 regulates autophagosome formation via Rab11- and ULK1-positive recycling endosomes. *J. Cell Biol.* 197, 659-675. 10.1083/jcb.201111079 22613832 PMC3365497

[BIO013979C39] Low P., Varga A., Pircs K., Nagy P., Szatmari Z., Sass M. and Juhasz G. (2013). Impaired proteasomal degradation enhances autophagy via hypoxia signaling in Drosophila. *BMC Cell Biol.* 14, 29 10.1186/1471-2121-14-29 23800266 PMC3700814

[BIO013979C40] Manil-Segalen M., Lefebvre C., Culetto E. and Legouis R. (2012). Need an ESCRT for autophagosomal maturation? *Commun. Integr. Biol.* 5, 566-571. 10.4161/cib.21522 23336026 PMC3541323

[BIO013979C41] Mari M., Griffith J., Rieter E., Krishnappa L., Klionsky D. J. and Reggiori F. (2010). An Atg9-containing compartment that functions in the early steps of autophagosome biogenesis. *J. Cell Biol.* 190, 1005-1022. 10.1083/jcb.200912089 20855505 PMC3101592

[BIO013979C42] Mindell J. A. (2012). Lysosomal acidification mechanisms. *Annu. Rev. Physiol.* 74, 69-86. 10.1146/annurev-physiol-012110-142317 22335796

[BIO013979C43] Mizushima N., Levine B., Cuervo A. M. and Klionsky D. J. (2008). Autophagy fights disease through cellular self-digestion. *Nature* 451, 1069-1075. 10.1038/nature06639 18305538 PMC2670399

[BIO013979C44] Mousavi S. A., Kjeken R., Berg T. O., Seglen P. O., Berg T. and Brech A. (2001). Effects of inhibitors of the vacuolar proton pump on hepatic heterophagy and autophagy. *Biochim. Biophys. Acta* 1510, 243-257. 10.1016/S0005-2736(00)00354-0 11342162

[BIO013979C45] Mulakkal N. C., Nagy P., Takats S., Tusco R., Juhasz G. and Nezis I. P. (2014). Autophagy in Drosophila: from historical studies to current knowledge. *Biomed. Res. Int.* 2014, 273473 10.1155/2014/273473 24949430 PMC4052151

[BIO013979C46] Nagy P., Varga A., Pircs K., Hegedus K. and Juhasz G. (2013). Myc-driven overgrowth requires unfolded protein response-mediated induction of autophagy and antioxidant responses in Drosophila melanogaster. *PLoS Genet.* 9, e1003664 10.1371/journal.pgen.1003664 23950728 PMC3738540

[BIO013979C47] Nagy P., Hegedus K., Pircs K., Varga A. and Juhasz G. (2014). Different effects of Atg2 and Atg18 mutations on Atg8a and Atg9 trafficking during starvation in Drosophila. *FEBS Lett.* 588, 408-413. 10.1016/j.febslet.2013.12.012 24374083 PMC3928829

[BIO013979C48] Nakatogawa H., Suzuki K., Kamada Y. and Ohsumi Y. (2009). Dynamics and diversity in autophagy mechanisms: lessons from yeast. *Nat. Rev. Mol. Cell Biol.* 10, 458-467. 10.1038/nrm2708 19491929

[BIO013979C49] Ohsumi Y. (2014). Historical landmarks of autophagy research. *Cell Res.* 24, 9-23. 10.1038/cr.2013.169 24366340 PMC3879711

[BIO013979C50] Orsi A., Razi M., Dooley H. C., Robinson D., Weston A. E., Collinson L. M. and Tooze S. A. (2012). Dynamic and transient interactions of Atg9 with autophagosomes, but not membrane integration, are required for autophagy. *Mol. Biol. Cell* 23, 1860-1873. 10.1091/mbc.E11-09-0746 22456507 PMC3350551

[BIO013979C100] Ouimet M. (2013). Autophagy in obesity and atherosclerosis: Interrelationships between cholesterol homeostasis, lipoprotein metabolism and autophagy in macrophages and other systems. *Biochim. Biophys. Acta.* 1831, 1124-1133. 10.1016/j.bbalip.2013.03.007 23545567

[BIO013979C101] Pan M., Maitin V., Parathath S., Andreo U., Lin S. X., St Germain C., Yao Z., Maxfield F. R., Williams K. J. and Fisher E. A. (2008). Presecretory oxidation, aggregation, and autophagic destruction of apoprotein-B: a pathway for late-stage quality control. *Proc. Natl. Acad. Sci. U S A* 105, 5862-5867. 10.1073/pnas.0707460104 18391222 PMC2311371

[BIO013979C102] Papinski D. and Kraft C. (2014). Atg1 kinase organizes autophagosome formation by phosphorylating Atg9. *Autophagy* 10, 1338-1340. 10.4161/auto.28971 24905091 PMC4203558

[BIO013979C51] Papinski D., Schuschnig M., Reiter W., Wilhelm L., Barnes C. A., Maiolica A., Hansmann I., Pfaffenwimmer T., Kijanska M., Stoffel I. et al. (2014). Early steps in autophagy depend on direct phosphorylation of Atg9 by the Atg1 kinase. *Mol. Cell* 53, 471-483. 10.1016/j.molcel.2013.12.011 24440502 PMC3978657

[BIO013979C52] Pircs K., Nagy P., Varga A., Venkei Z., Erdi B., Hegedus K. and Juhasz G. (2012). Advantages and limitations of different p62-based assays for estimating autophagic activity in Drosophila. *PLoS ONE* 7, e44214 10.1371/journal.pone.0044214 22952930 PMC3432079

[BIO013979C53] Popovic D. and Dikic I. (2014). TBC1D5 and the AP2 complex regulate ATG9 trafficking and initiation of autophagy. *EMBO Rep.* 15, 392-401. 10.1002/embr.201337995 24603492 PMC3989670

[BIO013979C54] Pulipparacharuvil S., Akbar M. A., Ray S., Sevrioukov E. A., Haberman A. S., Rohrer J. and Kramer H. (2005). Drosophila Vps16A is required for trafficking to lysosomes and biogenesis of pigment granules. *J. Cell Sci.* 118, 3663-3673. 10.1242/jcs.02502 16046475

[BIO013979C55] Puri C., Renna M., Bento C. F., Moreau K. and Rubinsztein D. C. (2013). Diverse autophagosome membrane sources coalesce in recycling endosomes. *Cell* 154, 1285-1299. 10.1016/j.cell.2013.08.044 24034251 PMC3791395

[BIO013979C56] Ragusa M. J., Stanley R. E. and Hurley J. H. (2012). Architecture of the Atg17 complex as a scaffold for autophagosome biogenesis. *Cell* 151, 1501-1512. 10.1016/j.cell.2012.11.028 23219485 PMC3806636

[BIO013979C57] Ravikumar B., Moreau K., Jahreiss L., Puri C. and Rubinsztein D. C. (2010). Plasma membrane contributes to the formation of pre-autophagosomal structures. *Nat. Cell Biol.* 12, 747-757. 10.1038/ncb2078 20639872 PMC2923063

[BIO013979C58] Reggiori F. and Tooze S. A. (2012). Autophagy regulation through Atg9 traffic. *J. Cell Biol.* 198, 151-153. 10.1083/jcb.201206119 22826119 PMC3410426

[BIO013979C103] Richards P., Didszun C., Campesan S., Simpson A., Horley B., Young K. W., Glynn P., Cain K., Kyriacou C. P., Giorgini F. et al. (2011). Dendritic spine loss and neurodegeneration is rescued by Rab11 in models of Huntington's disease. *Cell Death Differ.* 18, 191-200. 10.1038/cdd.2010.127 21217767 PMC3131896

[BIO013979C59] Rothstein E. C., Nauman M., Chesnick S. and Balaban R. S. (2006). Multi-photon excitation microscopy in intact animals. *J. Microsc.* 222, 58-64. 10.1111/j.1365-2818.2006.01570.x 16734715 PMC1473170

[BIO013979C60] Rubinsztein D. C., Shpilka T. and Elazar Z. (2012). Mechanisms of autophagosome biogenesis. *Curr. Biol.* 22, R29-R34. 10.1016/j.cub.2011.11.034 22240478

[BIO013979C61] Rusten T. E., Lindmo K., Juhasz G., Sass M., Seglen P. O., Brech A. and Stenmark H. (2004). Programmed autophagy in the Drosophila fat body is induced by ecdysone through regulation of the PI3K pathway. *Dev. Cell* 7, 179-192. 10.1016/j.devcel.2004.07.005 15296715

[BIO013979C62] Rusten T. E., Rodahl L. M. W., Pattni K., Englund C., Samakovlis C., Dove S., Brech A. and Stenmark H. (2006). Fab1 phosphatidylinositol 3-phosphate 5-kinase controls trafficking but not silencing of endocytosed receptors. *Mol. Biol. Cell* 17, 3989-4001. 10.1091/mbc.E06-03-0239 16837550 PMC1556381

[BIO013979C63] Rusten T. E., Vaccari T., Lindmo K., Rodahl L. M. W., Nezis I. P., Sem-Jacobsen C., Wendler F., Vincent J.-P., Brech A., Bilder D. et al. (2007). ESCRTs and Fab1 regulate distinct steps of autophagy. *Curr. Biol.* 17, 1817-1825. 10.1016/j.cub.2007.09.032 17935992

[BIO013979C64] Saitoh T., Fujita N., Hayashi T., Takahara K., Satoh T., Lee H., Matsunaga K., Kageyama S., Omori H., Noda T. et al. (2009). Atg9a controls dsDNA-driven dynamic translocation of STING and the innate immune response. *Proc. Natl. Acad. Sci. USA* 106, 20842-20846. 10.1073/pnas.0911267106 19926846 PMC2791563

[BIO013979C65] Satoh A. K., O'Tousa J. E., Ozaki K. and Ready D. F. (2005). Rab11 mediates post-Golgi trafficking of rhodopsin to the photosensitive apical membrane of Drosophila photoreceptors. *Development* 132, 1487-1497. 10.1242/dev.01704 15728675

[BIO013979C66] Savina A., Fader C. M., Damiani M. T. and Colombo M. I. (2005). Rab11 promotes docking and fusion of multivesicular bodies in a calcium-dependent manner. *Traffic* 6, 131-143. 10.1111/j.1600-0854.2004.00257.x 15634213

[BIO013979C67] Schroder B. A., Wrocklage C., Hasilik A. and Saftig P. (2010). The proteome of lysosomes. *Proteomics* 10, 4053-4076. 10.1002/pmic.201000196 20957757

[BIO013979C68] Sekito T., Kawamata T., Ichikawa R., Suzuki K. and Ohsumi Y. (2009). Atg17 recruits Atg9 to organize the pre-autophagosomal structure. *Genes Cells* 14, 525-538. 10.1111/j.1365-2443.2009.01299.x 19371383

[BIO013979C69] Settembre C., Fraldi A., Rubinsztein D. C. and Ballabio A. (2008a). Lysosomal storage diseases as disorders of autophagy. *Autophagy* 4, 113-114. 10.4161/auto.5227 18000397

[BIO013979C70] Settembre C., Fraldi A., Jahreiss L., Spampanato C., Venturi C., Medina D., de Pablo R., Tacchetti C., Rubinsztein D. C. and Ballabio A. (2008b). A block of autophagy in lysosomal storage disorders. *Hum. Mol. Genet.* 17, 119-129. 10.1093/hmg/ddm289 17913701

[BIO013979C71] Shandala T., Woodcock J. M., Ng Y., Biggs L., Skoulakis E. M. C., Brooks D. A. and Lopez A. F. (2011). Drosophila 14-3-3epsilon has a crucial role in anti-microbial peptide secretion and innate immunity. *J. Cell Sci.* 124, 2165-2174. 10.1242/jcs.080598 21670199

[BIO013979C72] Shen H.-M. and Mizushima N. (2014). At the end of the autophagic road: an emerging understanding of lysosomal functions in autophagy. *Trends Biochem. Sci.* 39, 61-71. 10.1016/j.tibs.2013.12.001 24369758

[BIO013979C73] Shibutani S. T. and Yoshimori T. (2014). A current perspective of autophagosome biogenesis. *Cell Res.* 24, 58-68. 10.1038/cr.2013.159 24296784 PMC3879706

[BIO013979C74] Singh R. and Cuervo A. M. (2011). Autophagy in the cellular energetic balance. *Cell Metab.* 13, 495-504. 10.1016/j.cmet.2011.04.004 21531332 PMC3099265

[BIO013979C75] Singh R., Kaushik S., Wang Y., Xiang Y., Novak I., Komatsu M., Tanaka K., Cuervo A. M. and Czaja M. J. (2009). Autophagy regulates lipid metabolism. *Nature* 458, 1131-1135. 10.1038/nature07976 19339967 PMC2676208

[BIO013979C76] Spowart J. and Lum J. J. (2010). Opening a new DOR to autophagy. *EMBO Rep.* 11, 4-5. 10.1038/embor.2009.265 20033084 PMC2816631

[BIO013979C77] Suzuki K., Kirisako T., Kamada Y., Mizushima N., Noda T. and Ohsumi Y. (2001). The pre-autophagosomal structure organized by concerted functions of APG genes is essential for autophagosome formation. *EMBO J.* 20, 5971-5981. 10.1093/emboj/20.21.5971 11689437 PMC125692

[BIO013979C78] Suzuki K., Kubota Y., Sekito T. and Ohsumi Y. (2007). Hierarchy of Atg proteins in pre-autophagosomal structure organization. *Genes Cells* 12, 209-218. 10.1111/j.1365-2443.2007.01050.x 17295840

[BIO013979C79] Takahashi Y., Meyerkord C. L., Hori T., Runkle K., Fox T. E., Kester M., Loughran T. P. and Wang H.-G. (2011). Bif-1 regulates Atg9 trafficking by mediating the fission of Golgi membranes during autophagy. *Autophagy* 7, 61-73. 10.4161/auto.7.1.14015 21068542 PMC3039731

[BIO013979C80] Takats S., Nagy P., Varga A., Pircs K., Karpati M., Varga K., Kovacs A. L., Hegedus K. and Juhasz G. (2013). Autophagosomal Syntaxin17-dependent lysosomal degradation maintains neuronal function in Drosophila. *J. Cell Biol.* 201, 531-539. 10.1083/jcb.201211160 23671310 PMC3653357

[BIO013979C81] Tamai K., Tanaka N., Nara A., Yamamoto A., Nakagawa I., Yoshimori T., Ueno Y., Shimosegawa T. and Sugamura K. (2007). Role of Hrs in maturation of autophagosomes in mammalian cells. *Biochem. Biophys. Res. Commun.* 360, 721-727. 10.1016/j.bbrc.2007.06.105 17624298

[BIO013979C82] Tang H.-W., Wang Y.-B., Wang S.-L., Wu M.-H., Lin S.-Y. and Chen G.-C. (2011). Atg1-mediated myosin II activation regulates autophagosome formation during starvation-induced autophagy. *EMBO J.* 30, 636-651. 10.1038/emboj.2010.338 21169990 PMC3041946

[BIO013979C83] Tang H.-W., Liao H.-M., Peng W.-H., Lin H.-R., Chen C.-H. and Chen G.-C. (2013). Atg9 interacts with dTRAF2/TRAF6 to regulate oxidative stress-induced JNK activation and autophagy induction. *Dev. Cell* 27, 489-503. 10.1016/j.devcel.2013.10.017 24268699

[BIO013979C84] Thumm M., Egner R., Koch B., Schlumpberger M., Straub M., Veenhuis M. and Wolf D. H. (1994). Isolation of autophagocytosis mutants of Saccharomyces cerevisiae. *FEBS Lett.* 349, 275-280. 10.1016/0014-5793(94)00672-5 8050581

[BIO013979C85] Tsukada M. and Ohsumi Y. (1993). Isolation and characterization of autophagy-defective mutants of Saccharomyces cerevisiae. *FEBS Lett.* 333, 169-174. 10.1016/0014-5793(93)80398-E 8224160

[BIO013979C86] Vaccari T., Rusten T. E., Menut L., Nezis I. P., Brech A., Stenmark H. and Bilder D. (2009). Comparative analysis of ESCRT-I, ESCRT-II and ESCRT-III function in Drosophila by efficient isolation of ESCRT mutants. *J. Cell Sci.* 122, 2413-2423. 10.1242/jcs.046391 19571114 PMC2704878

[BIO013979C87] Vanlandingham P. A. and Ceresa B. P. (2009). Rab7 regulates late endocytic trafficking downstream of multivesicular body biogenesis and cargo sequestration. *J. Biol. Chem.* 284, 12110-12124. 10.1074/jbc.M809277200 19265192 PMC2673280

[BIO013979C104] Wang J., Menon S., Yamasaki A., Chou H. T., Walz T., Jiang Y. and Ferro-Novick S. (2013). Ypt1 recruits the Atg1 kinase to the preautophagosomal structure. *Proc. Natl. Acad. Sci. U S A* 110, 9800-9805. 10.1073/pnas.1302337110 23716696 PMC3683756

[BIO013979C88] Webber J. L. and Tooze S. A. (2010). New insights into the function of Atg9. *FEBS Lett.* 584, 1319-1326. 10.1016/j.febslet.2010.01.020 20083107

[BIO013979C89] Wirth M., Joachim J. and Tooze S. A. (2013). Autophagosome formation--the role of ULK1 and Beclin1-PI3KC3 complexes in setting the stage. *Semin. Cancer Biol.* 23, 301-309. 10.1016/j.semcancer.2013.05.007 23727157

[BIO013979C105] Wucherpfennig T., Wilsch-Brauninger M. and Gonzalez-Gaitan M. (2003). Role of Drosophila Rab5 during endosomal trafficking at the synapse and evoked neurotransmitter release. *J. Cell Biol.* 161, 609-624. 10.1083/jcb.200211087 12743108 PMC2172938

[BIO013979C90] Yamada T., Carson A. R., Caniggia I., Umebayashi K., Yoshimori T., Nakabayashi K. and Scherer S. W. (2005). Endothelial nitric-oxide synthase antisense (NOS3AS) gene encodes an autophagy-related protein (APG9-like2) highly expressed in trophoblast. *J. Biol. Chem.* 280, 18283-18290. 10.1074/jbc.M413957200 15755735

[BIO013979C91] Yamamoto H., Kakuta S., Watanabe T. M., Kitamura A., Sekito T., Kondo-Kakuta C., Ichikawa R., Kinjo M. and Ohsumi Y. (2012). Atg9 vesicles are an important membrane source during early steps of autophagosome formation. *J. Cell Biol.* 198, 219-233. 10.1083/jcb.201202061 22826123 PMC3410421

[BIO013979C92] Young A. R. J., Chan E. Y. W., Hu X. W., Kochl R., Crawshaw S. G., High S., Hailey D. W., Lippincott-Schwartz J. and Tooze S. A. (2006). Starvation and ULK1-dependent cycling of mammalian Atg9 between the TGN and endosomes. *J. Cell Sci.* 119, 3888-3900. 10.1242/jcs.03172 16940348

[BIO013979C93] Yu L., McPhee C. K., Zheng L., Mardones G. A., Rong Y., Peng J., Mi N., Zhao Y., Liu Z., Wan F. et al. (2010). Termination of autophagy and reformation of lysosomes regulated by mTOR. *Nature* 465, 942-946. 10.1038/nature09076 20526321 PMC2920749

[BIO013979C94] Zhang X. D., Qi L., Wu J. C. and Qin Z. H. (2013). DRAM1 regulates autophagy flux through lysosomes. *PLoS ONE* 8, e63245 10.1371/journal.pone.0063245 23696801 PMC3656954

